# *Eucalyptus viminalis* leaf extract alters the productivity and blood parameters of healthy broiler chickens

**DOI:** 10.14202/vetworld.2020.2673-2680

**Published:** 2020-12-16

**Authors:** G. K. Duskaev, O. V. Kvan, Sh. G. Rakhmatullin

**Affiliations:** Department for Feeding Agricultural Animals and Fodder Technology, Federal Research Centre of Biological Systems and Agrotechnologies of the Russian Academy of Sciences, Orenburg – 460 000, Russia

**Keywords:** broiler chickens, *Eucalyptus viminalis* leaf extract, gamma-octalactone, productivity, quorum sensing

## Abstract

**Background and Aim:**

As an alternative to natural and chemically synthesized direct-acting bactericides, there has been an increase in the use of plant extracts, which possess a set of phytochemicals with potential for microbial disease control; this is due to the spectrum of secondary metabolites present in extracts, which include phenolic compounds, quinones, flavonoids, alkaloids, terpenoids, and polyacetylenes. The biologically active substances within plant extracts, which perform protective functions for plant tissues, can have ambiguous effects on the animal body. Therefore, the aim of this study was to assess the ability of gamma-octalactone, isolated from *Eucalyptus viminalis* extract, to inhibit various LuxI/LuxR quorum-sensing (QS) systems in bacteria, and to evaluate its effect on broiler chickens.

**Materials and Methods:**

Phytochemical analysis of *E. viminalis* extract was performed. The ability of gamma-octalactone to inhibit QS was evaluated using four different LuxI/LuxR bacterial test systems. *In vivo* assessments were performed on one hundred and twenty 7-day-old broiler chickens (Arbor Acres cross), split into four groups of 30 chickens: 1. Control group: Basic diet (BD); 2. experimental Group I: BD + gamma-octalactone at a dosage of 0.05 ml/kg live weight/day; 3. experimental Group II: BD + gamma-octalactone at a dosage of 0.1 ml/kg live weight/day; and 4. experimental Group III: BD + gamma-octalactone at a dosage of 0.2 ml/kg live weight/day. Hematological blood parameters were assessed using an automatic hematological analyzer (URIT-2900 Vet Plus, URIT Medial Electronic Co., China) and an automatic biochemical analyzer (CS-T240, Dirui Industrial Co., Ltd., China). Statistical analyses were performed using SPSS Statistics Version 20 (IBM); averages (M), standard deviations (σ), and standard deviation errors (m) were calculated. Results with p≤0.05 were considered significant.

**Results:**

Based on the phytochemical analysis results, libraries of compounds with putative QS inhibitory properties were compiled. Gamma-octalactone exhibited a pronounced inhibitory effect on the LuxI/LuxR QS systems, characterized by EC_50_ values of 0.15-0.4 mM. In the *in vivo* portion of this study, broiler chicken live weights increased in all experimental groups, with the most significant increase in Group III (14.0%), in relation to the control group. Blood serum from the experimental group chickens had significantly higher levels of triglycerides and uric acid (p≤0.05), in comparison to the control group chickens. With respect to blood serum enzyme activity and antioxidant status indicators, the experimental group chickens had a higher level of gamma-glutamyl transferase, an enzyme associated with amino acid metabolism, than those in the control group; this increase was especially pronounced in Group III, with 37.0% increase (p≤0.05). Superoxide dismutase and catalase levels were higher in the experimental groups than the control group, corresponding to increases of 30.4-56.2% (p≤0.05), 33.3-83.3%, and 27.9-45.5% (p≤0.05) in Groups I, II, and III (p≤0.05), respectively. Morphological blood parameters did not display significant changes due to gamma-octalactone.

**Conclusion:**

According to the results of this *in vivo* study in broiler chickens, gamma-octalactone, isolated from *E. viminalis* leaf extract and supplied at a dosage of 0.2 ml/kg live weight/day, led to an increase in the activity of blood plasma digestive enzymes, increased live weight, and had a positive effect on lipid metabolism and antioxidant status.

## Introduction

As an alternative to natural and chemically ­synthesized direct-acting bactericides, there has been an increase in the use of approaches that aim to suppress a microorganism’s ability to parasitize and damage biological substrates by altering its physiology. More specifically, this approach involves the search and creation of quorum-sensing (QS) inhibitors in bacteria. QS inhibitors are considered an attractive alternative to traditional infection treatments, especially for infections caused by multiresistant strains of microorganisms [1]. In bacteria, QS is understood to be a density-dependent means of communication, mediated by small diffusing molecules (autoinducers) and, on reaching a threshold population density, leading to functional and morphological differentiation of its constituent cells [2]. At the same time, discovery of this phenomenon of the ­collective behavior of bacteria has enabled a fundamental reevaluation of many examples of the functional and morphological differentiation of prokaryotes, including the formation of virulence factors and biofilm, conjugation, and sporogenesis [3].

Medicinal plants offer a set of phytochemicals that have potential for microbial disease control; this is due to the spectrum of secondary metabolites present in extracts, which include phenolic compounds, quinones, flavonoids, alkaloids, terpenoids, and polyacetylenes [4,5]. For example, cis-cis-p-Menthenolide, extracted from *Mentha suaveolens* ssp*. insularis*, acts as an inhibitor of violacein production (*Chromobacterium violaceum*) and biofilm formation [6]; similar effects have been found for Terpinen-4-ol (*Melaleuca alternifolia* essential oil) [7], *Carum copticum* essential oil and phenols [8], d-limonene nanoemulsion on *Escherichia coli* [9], *Salvadora persica* L. and *Astilbe rivularis*, *Fragaria nubicola*, and *Osbeckia nepalensis* [10] methanolic extracts, and other medicinal plants [11].

As such, this has inspired many studies of the medicinal plant eucalyptus. For example, studies have noted the high efficiency of *Eucalyptus globulus* essential oil and 1,8-cineole against the development of biofilms formed by methicillin-resistant strains of bacteria [12]. Nonetheless, the biological effects of the active compounds of the various vegetative parts of eucalyptus have not been studied. Moreover, it is also necessary to study the effect of individual compounds of herbal extracts on poultry. Recent studies have shown that α-Pinene can modulate quorum sensitivity, potentiate antibiotics, and reduce *Campylobacter* colonization in broiler chickens [13].

The aim of this study was to investigate the effects of gamma-octalactone, isolated from *Eucalyptus viminalis* leaf extracts, on the inhibition of various variants of LuxI/LuxR QS systems in bacteria, in addition to its effect on broiler chickens.

## Materials and Methods

### Ethical approval

Poultry maintenance and procedures during the experiments met the requirements of the instructions and recommendations of Russian regulations (Order of the Ministry of Health of the USSR No. 755 of 12.08.1977) and “The Guide for Care and Use of Laboratory Animals” (National Academy Press, Washington, D.C., 1996).

### Study period and location

This experiment was conducted in the Laboratory of Biological Tests and Examinations at the Federal Scientific Center for Biological Systems and Agricultural Technologies of the Russian Academy of Sciences in February 2020.

### Phytochemical analysis of *E. viminalis* extract

Eucalyptus leaves were purchased from the apothecary and then identified by Deryabin D. G. from Federal Scientific Center for Biological Systems and Agricultural Technologies of the Russian Academy of Sciences. Eucalyptus leaf extract was prepared according to the following procedure: 30 g of crushed leaves (pharmaceutical form) were placed into a heat-resistant container and 300 ml (70°C) of distilled water was added. This mixture was heated in a water bath for 15 min, then filtered using ashless filter paper (70 mm). The filtrate was dried to obtain dry matter measurements at a temperature of 60°C.

Dry extract was redissolved in methanol before chromatography-mass spectrometry and then introduced into the analytical cell of the chromatograph using a Hamilton 1700 microsyringe. Analysis was performed on a gas chromatograph with a mass-selective detector (GQCMS 2010 Plus, Shimadzu, Japan) on an HP-5MS column. GCMS PostRun Analysis software (GCMS Solutions) was used to interpret the results. A set of spectra libraries, namely, CAS, NIST08, Mainlib, Wiley9, and DD2012 Lib, were used to identify compounds. The quantitative presence of individually identified components was estimated by its relative value (%), with a correlation of the peak area to the total area of the extract.

### Substance

Gamma-octalactone, Sigma-Aldrich.


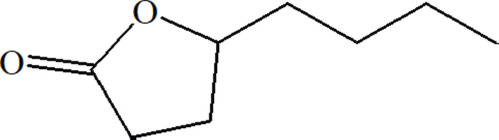


### Assessment of QS inhibition

Four different LuxI/LuxR bacterial test systems were used to determine the ability of gamma-octalactone to inhibit QS; each test system was induced by a specific acyl-homoserine lactone (AHL) and contained the corresponding LuxR-like receptor protein. Three of the test systems were based on *E. coli* JLD271, with a mutation in the *sdiA* gene responsible for the perception of exogenous AHL; this excluded interference with genetically engineered bioluminescent constructions cloned in the host strain [14]. In turn, the genetic constructs themselves were represented by plasmids with the gene sequences rhlR + rhlI :: luxCDABE, luxR + luxI :: luxCDABE, and lasR + lasI :: luxCDABE, specifically reacting to luminescence with C4-AGL (N-butanoyl-L-homoserine lactone), C6-oxo-AHL (N-(oxohexanoyl)-L-homoserine lactone), and C12-oxo-AHL (N-(oxododecanoyl)-L-homoserine lactone), respectively. The fourth test system encompassed *C. violaceum* NCTC 13274, with the insertion of the Tn5 transposon into the *cviI* gene. This gene is responsible for synthesizing C6-AHL (N-hexanoyl-L-homoserine lactone) and maintaining the functionally active *cviR* gene and the receptor protein encoded by it, which is responsible for the perception of this autoinductor [15]. Due to its genetic organization, in the presence of exogenously introduced Cg-AHL, this strain forms the blue-violet pigment violacein, which can be optically registered (575 nm).

The ability of gamma-octalactone to inhibit the aforementioned QS systems was assessed through a series of 2-fold dilutions into a liquid nutrient media containing bacterial biotest and the corresponding AHL. Inhibitory effects were assessed through EC_50_ values, expressed in mM. EC_50_ corresponds to the concentration of gamma-octalactone that suppresses bioluminescence or prevents the formation of violacein pigment by 50% of the maximum possible pronounced effect, calculated relative to the following corresponding controls: 0% in the absence of AHL; 100% in the presence of only AHL.

### Experimental design

The study was designed as a 4 × 4 Latin square with three replications.

Experimental studies were carried out on one hundred and twenty 7-day-old broiler chickens (Arbor Acres), divided into four groups of 30 chickens: 1. Control group: Basic diet (BD); 2. experimental Group I: BD + gamma-octalactone at a dosage of 0.05 ml/kg live weight/day; 3. experimental Group II: BD + gamma-octalactone at a dosage of 0.1 ml/kg live weight/day; and 4. experimental Group III: BD + gamma-octalactone at a dosage of 0.2 ml/kg live weight/day (Table-1). Chickens were feed and watered according to All-Russian Research Institute of Poultry Farming recommendations.

**Table-1:** Ingredients and nutrient level of basal diets.

Attributes	Starter (7-28 days)	Finisher (29-42 days)
	
	Control, I, II, III	Control, I, II, III
Ingredient composition (%)		
Wheat	47	42.0
Barley	2.6	0.3
Corn	7.5	22.0
Soybean meal (46% CP)	25.0	15.0
Sunflower meal (38% CP)	7.0	10.0
Sunflower oil	5.0	5.0
Di-Ca phosphate	1.6	1.4
Mel stern	0.9	1.5
Limestone	0.5	0.3
Salt	0.36	0.2
DL-Methionine	0.18	0.16
L-Lysine	0.35	0.17
Sodium bicarbonate	0.1	0.2
Vitamin-mineral premixa^a^	2.0	2.0
	100	100
Calculated nutrients		
Metabolizable energy (kkal/100 g)	296	302.0
Crude protein	22.0	19.7
Crude fat	6.68	7.05
Crude fiber	4.43	4.2
Methionine + cysteine	0.87	0.79
Lysine	1.35	0.96
Ca	0.95	1.01
Available phosphorus	0.54	0.48

a Supplied following per kilogram of diet: Vitamin A: 7000 IU; Vitamin D3: 800.0 IU; Vitamin E: 9 IU; Vitamin K3: 1.1 mg; thiamine: 0.7 mg; riboflavin: 3.0 mg; Vitamin B6: 1 mg; Vitamin B12: 0.01 mg; Vitamin C: 50 mg; Mn: 23 mg; Fe: 17 mg; Zn: 11 mg; Cu: 2.5 mg; I: 0.4 mg; Se: 0.2 mg. Ca=Calcium

At the end of each period (starter and finisher), broiler body weights were assessed. At the end of the experiment, feed consumption and mortality were assessed. Body weight gain, daily feed intake, and feed conversion ratio were calculated for each group. At the end of the experiment (42 days of age), 10 birds with an average weight were selected, and their blood was sampled through wing vein puncture while still alive.

### Hematological analysis

Morphological blood parameters (hemoglobin, hematocrit, erythrocyte [red blood count], and leukocyte [white blood cell (WBC)]) were determined using an automatic hematological analyzer (URIT-2900 Vet Plus, URIT Medial Electronic Co., China). Blood serum biochemical analysis assessed glucose (GLU), total protein (TP), albumin (ALB), uric acid (UA), urea (UREA), bilirubin, creatinine, total cholesterol and its fractions (i.e., high-density cholesterol and triacylglycerols), alkaline phosphatase (ALP; EC 3.1.3.1), alanine aminotransferase (ALT; EC 2.6.1.2), aspartate aminotransferase (AST; EC 2.6.1.1), lactate dehydrogenase (EC 1.1.1.27), phosphorus, calcium, and magnesium levels; this analysis was performed with an automatic biochemical analyzer (CS-T240, Dirui Industrial Co., Ltd., China) using commercial veterinary medicine biochemical kits (DiaVetTest, Russia) and Randox biochemical kits (Laboratories Limited, UK).

### Statistical analysis

Statistical analyses were performed using SPSS Statistics Version 20 (IBM Corp., NY, USA). Average values (M), standard deviations (σ), and standard deviation errors (m) were calculated. Results with p≤0.05 were considered statistically significant.

## Results

### Phytochemical analysis of the extract

The analysis accounted for incubation time in the column and revealed a relative content, estimated according to peak area in the chromatogram, probability of identification (not <85%), and physicochemical characteristics that suggested the presence of biological properties in substances. Libraries of compounds with putative QS inhibiting properties were identified: 2.4-dihydroxy-2.5-dimethylfuran-3-one; 1-Methyl-4-(1-methylethyl) benzene; 3-Hydroxy-2-methyl-4H-pyran-4-one; 3,4-dihydroxyoxolan-2-one; 2-ethyl-5-methyl-1.3.2-dioxaborolan-4-one; gamma.hexalactone; gamma.lactone; 1.4: 3.6-dianhydro-2.5-di-O-nitro-D-glucitol; 4-hydroxy-3-methyl-6-propan-2-ylcyclohex-2-en-1-one; 2-hydroxy-3-phenylpropanoic acid; shikimic acid; loliolide; rosifoliol; and 4H-1-Benzopyran -4-one, 5.7-dihydroxy-2-methyl.

### Assessment of QS inhibition

Gamma-octalactone exhibited a pronounced inhibitory effect on all investigated LuxI/LuxR QS systems; this effect was characterized by EC_50_ values of 0.15-0.4 mM (Table-2). As such, an *in vivo* assessment of the effect of gamma-octalactone on the body weight of broiler chickens was warranted.

**Table-2:** Evaluation of the inhibitory effect of gamma-octalactone on various variants of the LuxI/LuxR type “quorum-sensing” system in bacteria, estimated by EC_50_ values.

Variants of quorum-sensing systems (AHL/LuxR-like receptor protein)	Gamma-octalactone
C4-AHL/RhlR	0.4 mM
C6-oxo-AHL/LuxR	0.44 mM
C12-oxo-AHL/LasR	0.16 mM
C6-AHL/CviR	0.15 mM

### Growth performance

Across the whole experiment, live weights increased in all experimental groups. Significant changes in broiler live weights were noted during the 1^st^ week of the experiment, during which live weights from experimental Group III broilers increased by 11.3-16.3% (p≤0.05) compared to those in the control group. A similar pattern was observed at the end of the experiment, during which live weights significantly increased by 14.0% in relation to the control group (Table-3). Broiler chickens from Group III also demonstrated an average daily gain increase of 18.3% (p≤0.05), and an absolute increase of 18.29% live weight (p≤0.05), in comparison to broilers in the control group. Broilers in Groups I, II, and III had lower feed intakes, amounting to 6.70-7.26% less than that of broilers in the control group.

**Table-3:** Productive indicator of broiler chickens, g/head.

Weeks	Group			

	Control	I group	II group	III group
Start of the experiment	340.00±6.6	340.00±7.7	340.00±8.5	340.00±9.3
1	612.80±12.5	667.60±13.0	676.40±21.6	682.00±24.6*
2	1049.60±32.3	1211.60±36.1	1215.20±50.1	1164.40±40.5
3	1682.00±37.4	1842.00±39.2	1852.40±39.3	1829.60±54.4
4	2273.20±49.0	2557.20±39.0	2546.80±41.8	2602.40±65.6
5	2896.00±88.3	3267.00±35.4	3288.00±74.1	3368.00±78.7
Average daily gain, g/head/day	73.03±5.8	83.46±4.5	84.3±4.9	86.39±5.4*
Absolute gain, g/head/exper.	2556.00±88.34	2921.0±31.71	2950.5±174.68	3023.5±82.60*
Amount of feed consumed, kg/head	4567.5±151.47	4836.9±53.69	4938.0±315.42	5020.86±135.34
Feed consumption per an increase of 1 kg of live masses, kg/head	1.79±0.058	1.66±0.018	1.67±0.107	1.66±0.045

* p≤0.05 in comparison with the control group

## Blood test results

Dietary doses of 0.05, 0.10, and 0.20 ml of gamma-octalactone/kg live weight/day had a direct impact on the hematological parameters of broiler chickens. At 42 days of age, there was a decrease in the ALB (p≤0.05) and urea (p≤0.05) levels in the blood serum of broiler chickens in experimental Group III, with an increase in triglycerides (p≤0.05) and UA (p≤0.05), in comparison with the control group. Similarly, broilers in Group I had increased levels of urea (p≤0.01), UA and triglycerides (p≤0.05), and decreased levels of cholesterol (by 25.9%, p≤0.05) relative to those in the control group (Table-4). Broilers in Group II had a 2.6-time decrease in urea (p≤0.01) and 1.6-time increase in UA (p≤0.05), in comparison to broilers in the control group.

**Table-4:** Blood biochemical parameters of broiler chickens (M ± m, n = 30, experiment in the conditions of vivarium).

Indicator	Group			

	Control	Group I	Group II	Group III
GLU, mmol/L	12.41±0.25	12.71±0.48	12.57±0.58	12.40±0.45
TP, g/L	41.87±1.18	39.22±2.03	39.09±1.07	37.86±1.82
ALB, g/L	18.00±1.00	15.67±1.67	16.67±0.67	14.67±0.33*
Total bilirubin, μmol/L	0.40±0.17	0.59±0.25	0.65±0.19	0.39±0.00
Direct bilirubin, μmol/L	0.34±0.05	0.45±0.04	0.34±0.03	0.45±0.04
Cholesterol, mmol/L	3.21±0.07	2.38±1.15*	3.49±0.07	3.44±0.44
Triglycerides, mmol/L	0.27±0.03	0.44±0.07*	0.29±0.04	0.42±0.04*
Urea, mmol/L	0.77±0.03	0.20±0.06**	0.30±0.10**	0.20±0.06**
CREAT, μmol/L	3.87±1.13	2.43±0.38	4.27±0.95	2.47±0.63
UA, μmol/L	79.13±9.81	126.37±13.81*	123.97±24.79*	102.93±28.92*
Calcium, μmol/L	4.15±0.09	4.22±0.10	3.54±0.04	4.82±0.49
P, mmol/L	4.10±0.14	4.16±0.10	4.30±0.08	4.34±0.04
Mg, mmol/L	1.31±0.05	1.25±0.08	1.29±0.02	1.52±0.16
Iron, μmol/L	11.73±1.05	13.63±1.98	10.93±1.39	10.97±2.00

* p≤0.05 in comparison with the control group.

** p≤0.01 in comparison with the control group. GLU=Glucose, TP=Total protein, ALB=Albumin, UA=Uric acid, CREAT=Creatinine, P=Phosphorus, Mg=Magnesium

In terms of blood serum enzyme activity and indicators of antioxidant status, in comparison to control broilers, the experimental group broilers demonstrated an increase in gamma-glutamyl-transferase, an enzyme associated with amino acid metabolism; this increase was especially notable in Group III broilers (37% increase, p≤0.05). Broilers in all experimental groups demonstrated a higher content of amylolytic enzyme (α-amylase, p≤0.05), whereas broilers in Groups II and III demonstrated a lower concentration of pancreatic amylase (p-amylase), in comparison to broilers from the control group. All experimental group broilers had higher levels of triglycerides and lipase enzyme, especially in Groups I and II (p≤0.05). Levels of superoxide dismutase and catalase, indicators of antioxidant status, were higher in the experimental groups than the control group, with differences of 30.4-56.2% (p≤0.05) and 33.3-83.3% in Groups I and II (p≤0.05). Conversely, levels of malondialdehyde, a marker of fat peroxidation and oxidative stress, were lower in e experimental group broilers (p≤0.05, Table-5). The morphological blood parameters of the experimental and control groups broiler chickens were similar (Tables-6 and 7).

**Table-5:** The activity of blood serum enzymes and indicators of the antioxidant status of broiler chickens (M±m, n=30, experiment under vivarium conditions).

Indicator	Group			

	Control	I	II	III
Alanine aminotransferase, U/L	13.8±3.32	16.0±1.85	16.8±1.88	15.5±1.76
Aspartate transferase, U/L	425.2±106.3	489.8±25.7	410.8±67.5	421.9±34.5
Gamma-glutamyltransferase, U/L	24.3±2.03	20.0±1.53	20.0±1.53	29.7±3.18
ALP, U/L	1752.3±163.8	1857.3±238.7	2092.0±46.5	2401.0±270.4*
α-Amylase, U/L	34.3±16.8	67.3±9.61*	52.7±4.06*	61.0±8.14*
p-Amylase, U/L	333.6±72.5	413.1±100.4	310.4±49.1	218.6±74.0
Lipase, U/L	5.03±2.51	14.40±4.35*	8.90±1.00*	5.83±0.86
Lactate dehydrogenase, U/L	1887.3±355.4	2181.3±368.2	1789.3±366.1	2121.7±121.5
Superoxide dismutase,%	41.7±2.25	54.42±5.76*	65.2±7.57*	57.8±3.58*
Malonic dialdehyde, μM/L	571.4±95.9	188.3±96.2*	212.7±64.1*	176.3±27.1*
Catalase, μM H_2_O_2_/lxmin	0.06±0.04	0.08±0.02*	0.05±0.01	0.11±0.06*

ALP=Alkaline phosphatase

**Table-6:** Morphological parameters of blood of broiler chickens (M±m, n=30, experiment in a vivarium).

Indicator	Group			

	Control	I	II	III
RBC, 10^12^/L	2.69±0.63	3.13±0.55	3.25±0.62	3.85±0.07
Hemoglobin, g/L	118.33±3.28	108.33±8.41	114.33±4.67	109.67±3.76
Packed cell volume/hematocrit, %	22.13±0.58	19.87±1.53	21.03±0.75	20.30±0.64
Mean cell volume, fl	109.87±1.42	110.93±0.09	110.20±1.05	109.97±1.19
Mean cell hemoglobin, pg	58.60±1.48	60.23±0.75	59.63±0.79	59.27±0.50
Mean cell hemoglobin concentration, g/L	534.33±6.39	544.67±6.69	543.00±4.62	539.67±1.86
Platelet count, 109/L	132.33±21.17	106.67±1.76	122.67±16.83	134.00±14.43

RBC=Red blood count

**Table-7:** The content of WBC (M ± m, n = 30, experiment in a vivarium).

Indicator	Group			

	Control	I	II	III
White cell count, 10^9^/L	48.23±0.93	40.77±1.33	51.33±4.03	45.17±2.82
Lymphocyte count, %	49.37±0.48	51.47±1.74	48.93±1.53	50.93±0.55
Monocyte count, %	8.47±0.23	7.93±0.18	9.13±0.43	8.60±0.26
Granulocytes, %	42.17±0.43	40.60±1.63	41.93±1.10	40.47±0.29

WBC=White blood cell

## Discussion

QS systems control the pathogenesis of living organisms, regulating gene expression, and enabling the development of new anti-infective agents without dependence on antibiotics. Anti-QS compounds are known to inhibit bacterial pathogenicity. More specifically, certain plant extracts, including phenolic compounds, quinones, flavonoids, alkaloids, terpenoids, and polyacetylenes, can effectively and safely inhibit QS. For example, McClean *et al*. [16] identified a clear relationship between *C. violaceum* and anti-QS activity.

Plant extracts that interact with the QS system of bacteria can form biofilms, which synthesize virulence factors or other compounds. Antibiotics kill or slow the growth of bacteria, and inhibitors sensitive to quorum or quorum quenchers weaken bacterial virulence. Studies have demonstrated that QS exists in *Pseudomonas aeruginosa*, *Staphylococcus aureus*, *Vibrio fischeri*, *Vibrio harveyi*, *E. coli*, and *Vibrio cholerae* [17].

*Senegalia nigrescens* crude extract, when used together with new terpenoids and flavonoids, has potential anti-QS activity [18]. *Melaleuca bracteata* essential oil can act as a potential antibacterial agent and QS inhibitor in pathogens, preventing and controlling bacterial contamination [19]. *S. persica* L. methanol extract demonstrates anti-QS effects at very low concentrations; this extract can be additionally used for the treatment of oral staphylococcal infections [20]. Extracts from three plants, *A. rivularis*, *F. nubicola*, and *O. nepalensis*, have demonstrated dose-dependent inhibition of violacein production, without negatively affecting bacterial growth, revealing QS inhibition properties [21]. *E. globulus* essential oil shows a higher activity against QS, even at a low concentration, than 1,8-cineole [22].

The poultry industry is developing at a fast pace; this industry provides the world’s population with an inexpensive, high-quality, and high-grade food product. The positive effect of medicinal plants on productive and economic indicators in poultry farming, and in particular the effect of medicinal plant mixtures on antioxidant status in poultry, is well known. The scientific literature contains limited data on the use of plant molecules in poultry nutrition, but the results of the current study will nonetheless be compared with available data on the effect of plant extracts on broiler chickens.

In broiler chickens, the dietary inclusion of eucalyptus powder with an antibiotic has been shown to lead to a significant increase in WBCs and a decrease in cholesterol levels [23]. Polyphenols, present in eucalyptus leaf extract, improve egg indicators (density, elastic deformation, gas permeability to air, and size of yolk) and laying hen meat ^­^quality; as in our study, this effect is due to an increase in enzymatic activity and antioxidants in the blood serum, which protects liver tissue from oxidative damage [24]. Mashayekhi *et al*. [25] found that eucalyptus leaf extract has antibacterial properties and can inhibit the growth of pathogenic bacteria and protozoa.

Ahlem *et al*. [26] studied the effect of plant extracts from three different medicinal plants (*Aristolochia ringens*, *Allium sativum*, and *Ocimum gratissimum*), but noted that they did not lead to significant changes in many indicators, such as TP, erythrocytes, ALT, and AST. We noted similar results in our study when using gamma-octalactone isolated from *E. viminalis* extract. The addition of thyme, mint, and eucalyptus to the diet of broiler chickens contributes to a significant increase in live weight; however, it also decreases the serum concentration of liver enzymes (e.g., ALT, AST, and ALP). In our study, at a dosage of 0.2 ml/kg live weight/day, we only noted non-significant decreases in AST, but did see a significant increase in live weight. Khalaji [27] noted that *E. globulus* extract lowers blood GLU level in diabetic animals and exhibits antioxidant activity, as demonstrated by an increase in catalase, superoxide dismutase, and glutathione peroxidase activity, and a decrease of lipid peroxidation levels in the liver and kidneys. In the current research, antioxidant activity was manifested by a significant increase in superoxide dismutase in all experimental groups and catalase in Groups I and II. Mustafa [28] evaluated the effect of *Camellia* L. plant extract in broiler chickens’ diets, noting a significant decrease in the total number of leukocytes, cholesterol concentration, and productivity as a result of the extract. Similar to the results of our study, Mashayekhi *et al*. [29] noted that *Eucalyptus camaldulensis* leaf extract increases body weight of broiler chickens, as well as the secretion of digestive enzymes, such as lipase and amylase. Damjanović-Vratnica *et al*. [30] found an antimicrobial effect of eucalyptus oil, especially against *Streptococcus pyogenes*, *E. coli*, *Candida albicans*, *S. aureus*, *Acinetobacter baumannii*, and *Klebsiella pneumoniae*; the authors suggest that this antimicrobial effect is due to the presence of monoterpenes and oxygenated monoterpenes, which are the main group of bactericidal molecules present in eucalyptus. Rehman *et al*. [31], after the introduction of eucalyptus oil to broilers’ drinking water, observed increases in body weight and feed conversion; however, Barbour *et al*. [32] noted an opposite result.

## Conclusion

The biologically active substances within plant extracts, which perform protective functions for plant tissues, can have ambiguous effects on the animal body. The results of our research indicate that the provision of gamma-octalactone, contained in *E. viminalis* leaf extract, led to changes in broiler chickens. More specifically, in broiler chickens, gamma-octalactone impacts digestive enzyme activity, as assessed through blood plasma, increases live weight, and positively affects lipid metabolism.

## Authors’ Contributions

GKD and ShGR equally designed and contributed to the experimentation. GKD and OVK wrote and edited the article. All authors read and approved the final manuscript.
